# Type 2 Diabetes Mellitus and Obstructive Sleep Apnoea Syndrome in the Elderly

**DOI:** 10.3390/healthcare13111266

**Published:** 2025-05-27

**Authors:** Lucía Ortega-Donaire, Sebastián Sanz-Martos, María Fernández-Martínez, Cristina Fernández-Martínez, Ganna Ovsyeyenko

**Affiliations:** 1 Department of Nursing, Faculty of Health Sciences, University of Jaen, 23071 Jaen, Spain; lortega@ujaen.es (L.O.-D.); cfmartin@ujaen.es (C.F.-M.); 2 061 Health Emergency Centre, Andalusian Health Service, 23071 Jaen, Spain; mefernan@ujaen.es; 3 Faculty of Health Sciences, University of Jaen, 23071 Jaen, Spain; go000002@red.ujaen.es

**Keywords:** obstructive sleep apnoea syndrome, type 2 diabetes, elderly, chronic disease

## Abstract

**Introduction**: Older people with sleep disturbances also have other chronic pathologies that may interfere with these disturbances. One of the comorbidities that is frequently present is type 2 diabetes. **Objective**: This research aims to find out whether type 2 diabetes mellitus present in elderly people affects the level of severity of obstructive sleep apnoea syndrome (OSAS). **Methodology**: A cross-sectional descriptive study was carried out on a sample of 134 elderly people who attended the Sleep Unit of Andalusia’s Door Hospital in Jaen, who were diagnosed with OSAS and classified according to severity. A total of 34 participants had a diagnosis of diabetes mellitus at the time of the study. **Results**: There were significant differences in the severity of obstructive sleep apnoea syndrome between participants with and without type 2 diabetes mellitus, with the former having higher scores (*p* < 0.01). Participants with a BMI that classified them as obese had more severe apnoea than those with a normal weight at the time of the study (*p* = 0.043). **Discussion**: This study, focused exclusively on older adults, demonstrates an association between type 2 diabetes mellitus and a greater severity of OSAS. Using polysomnography (PSG) as the gold standard, we identified a significant relationship between obesity and severe OSAS. Furthermore, the connection between OSAS, type 2 diabetes mellitus, and CPAP use highlights the importance of a comprehensive approach in this population.

## 1. Introduction

Obstructive sleep apnoea syndrome (OSAS) is characterised by recurrent episodes of upper airway collapse during sleep, resulting in apnoea (airflow blocked for at least 10 s) and hypopnoea (a discernible reduction in flow signal greater than 30% and less than 90% lasting more than 10 s and accompanied by desaturation of 3% or more, a microarousal detected on an EEG, or both) associated with daytime symptoms, particularly excessive sleepiness. OSAS is a highly prevalent and often unrecognised disease in the elderly with important cardiovascular and metabolic consequences. Obesity and advanced age have been identified as key predisposing factors in the pathophysiology of OSAS, contributing to airway obstruction and exacerbating its systemic effects. Severely affected patients experience more than 30 abnormal respiratory events per hour, with repeated hypoxia, and altered sleep architecture characterised by frequent awakenings and increased respiratory effort. The gold standard for diagnosing OSAS is a polysomnography (PSG) study performed in a sleep laboratory. The main treatment used to manage OSAS is continuous positive airway pressure (CPAP), which is a non-invasive device that helps regulate upper airway pressures during sleep [1].

Another highly prevalent disease is diabetes, which is a chronic disease caused by impaired glucose metabolism. Due to high morbidity and mortality, diabetes is considered a public health problem that severely affects the quality of life of patients. The latest estimates show that the global prevalence of type 2 diabetes is 382 million (8.3%), and it is expected to increase to 592 million (10.1%) by 2035 [2]. The reasons for this growth include the ageing of the population and improved diagnostic standards [3].

OSAS and type 2 diabetes are comorbidities with important clinical, epidemiological, and public health implications. Studies over the past 20 years suggest that OSAS, through the effects of intermittent hypoxemia, elevated sympathetic nervous activity, sleep fragmentation, and low amounts of slow-wave sleep, may contribute to the development of insulin resistance, glucose intolerance, and type 2 diabetes [1,4,5,6,7].

Regarding the severity of OSAS, a recent prospective population-based study with a 4-year follow-up period found that the presence of moderate to severe OSAS is an important risk factor for developing incident diabetes. Furthermore, the dysregulation of the hypothalamic–pituitary–adrenal axis in patients with severe OSAS has also been described as an additional factor in metabolic disturbances [8]. Moreover, type 2 diabetes may increase one’s susceptibility to OSAS or accelerate the progression of OSAS severity, possibly through the development of peripheral neuropathy and abnormal neural control of ventilation and upper airways [3,9].

The prevalence of OSAS in patients with type 2 diabetes is in the range of 50–80% [9]. As known and highly prevalent pathologies, OSAS and type 2 diabetes share similar risk factors, such as advanced age, visceral adiposity, and obesity; thus, some patients present both pathologies simultaneously [2,3]. The prevalence of OSAS in people over 65 years of age with diabetes mellitus is 60% [10].

In older people, there is a lack of scientific evidence to corroborate the relationship between these two variables. Older people, compared to younger adults, have more recurrent and intermittent hypoxia, a higher frequency of spontaneous arousal (a lower threshold for stimulation), and decreased upper airway muscle reflexes to negative pressure [9,11]. The occurrence of OSAS with age may be due to the four physiological factors considered important in the pathogenesis of OSAS, such as poor upper airway anatomy (highly collapsible airways), ineffective upper airway dilator muscle activity, a low respiratory activation threshold, or an unstable ventilatory control system. These factors, which can occur in the normal ageing process in healthy individuals, suggest that OSAS may be caused by different mechanisms in older and younger individuals [11].

Although further research is needed to elucidate the mechanisms underlying the bidirectional association between these two disorders, their frequent coexistence should prompt the adoption in clinical practice of screening for patients presenting with one disorder or the other. The early detection of OSAS in patients with type 2 diabetes and screening for metabolic abnormalities in patients with OSAS may reduce the risk of cardiovascular disease and improve the quality of life of people with these chronic diseases [4].

Therefore, the present research aims to determine whether type 2 diabetes mellitus present in elderly people affects the level of severity of obstructive sleep apnoea syndrome, expecting to find that elderly people with type 2 diabetes mellitus will have a higher degree of OSAS.

## 2. Materials and Methods

### 2.1. Design

A cross-sectional study was conducted. This article follows STROBE (Strengthening the Reporting of Observational Studies in Epidemiology) guidelines [12].

### 2.2. Sample and Settings

The inclusion criteria used for people to take part in this research were as follows: people over 65 years of age with a suspected diagnosis of obstructive sleep apnoea and referred to the specialised sleep unit of Andalucia’s Door Hospital in the province of Jaen. A convenience sample was taken. The main exclusion criteria were being under 65 years of age and not having previous glycaemic control that would lead to a diagnosis of type 2 diabetes mellitus. The participants included in this study signed the informed consent form before the beginning of the study.

### 2.3. Data Collection

Study participants attended the Sleep Unit of Puerta de Andalucía Hospital between 1 January and 31 December 2023. These participants attended the unit to be diagnosed with OSAS for the first time, referred by their Primary Care physician after observing symptoms consistent with the pathology, or to review their CPAP treatment, having been previously diagnosed with OSAS at this unit. The diagnosis of type 2 diabetes mellitus was made by obtaining data from the health history of the Andalusian Health Service [13].

OSAS was defined using the PSG test, which considers neurophysiological and cardiorespiratory variables and provides the Apnoea–Hypopnea Index (AHI), which determines the severity of the disease. When a person experiences 5 to 15 abnormal respiratory events/hour, their case is considered mild; when 16 to 29 abnormal respiratory events/hour are recorded, their case is considered moderate; and if more than 30 abnormal respiratory events/hour are recorded, their case is considered severe. Participants with fewer than 5 abnormal respiratory events/hour were classified as not having apnoea [14,15].

Type 2 diabetes mellitus was determined using the standardized test of glycated haemoglobin fraction (HbA1c) ≥6.5%. This parameter was determined from blood samples obtained from participants after a minimum fast of 8 h or by plasma glucose two hours after the oral tolerance test, with hyperglycaemia being considered a figure equal to or greater than 200 mg/dL [13].

The variables that could affect the results, based on biological plausibility and the previous literature, were age and sex, which were obtained from the medical history. Another variable taken into account was the Body Mass Index (BMI), which was obtained using a scale and a measuring tape, and calculated through the mathematical formula kg/m^2^. BMI was classified as follows: <18.4 kg/m^2^ underweight; 18.5–24.9 kg/m^2^ normal weight; 25–29.9 kg/m^2^ overweight; and >30 kg/m^2^ obese [15]

The results were classified according to the degree of severity of OSAS presented by the participant: mild OSAS, moderate OSAS, severe OSAS, or no OSAS (when AHI was under 5 incidents of apnoea–hypopnea/hour) and the presence or absence of a diagnosis of type 2 diabetes mellitus.

### 2.4. Data Analysis

Firstly, a descriptive analysis of the different variables considered in the study was carried out. In the case of qualitative variables, frequencies were used, and for quantitative variables, measures of central tendency (mean or median, depending on whether or not the distribution of the variables was normal) and dispersion (standard deviation or range) were calculated.

The normality of the distribution of the variables was assessed using the Kolmogorov–Smirnov test (with Lilliefors correction). Since the assumption of normality was not met, non-parametric tests (Mann–Whitney’s U) were used, depending on the number of categories of the independent variables of contrasts.

Analyses were performed with Jasp statistical package version 0.19. A significance level of 5% was used for all tests. All data generated or analysed during this study are included in the published article.

### 2.5. Ethical Considerations

The elderly people were informed about the purpose of the study and the data we would extract from their medical history. Those who agreed to participate would become part of the research after reading and signing the informed consent form (LOD2018).

Participants were informed and agreed to have their data published in a scientific article.

## 3. Results

### 3.1. Descriptive Analysis

The total sample consisted of 134 people, of whom 58 (43.28%) were women and 76 (56.72%) were men. The mean age was 71.07 (SD: 5.41) years. Regarding anthropometric measurements, the sample had a mean BMI value of 32.42 kg/m^2^ (SD = 5.28 kg/m^2^). The results relating to the severity of OSAS and type 2 diabetes mellitus were adjusted by BMI.

The OSAS severity data showed that 43.28% of the participants had no OSAS (with an AHI less than five abnormal respiratory events/hour); 20.81% had mild OSAS (with an AHI between 5 and 14.9 abnormal respiratory events/hour); 15.67% had moderate OSAS (with an AHI between 15 and 29.9 abnormal respiratory events/hour); and 20.15% had severe OSAS (with an AHI greater than or equal to 30 abnormal respiratory events/hour). With regard to the sociodemographic variables collected in this study, we found more men without OSAS (62.1%) than women (37.9%), and more women with severe OSAS (55.6%) than men (44.4%), although there were no significant relationships or differences (*p* = 0.505). Of the total number of people with type 2 diabetes mellitus, 17.6% did not suffer from OSAS, 20.6% suffered from it mildly, 20.6% moderately, and 41.2% severely. In Table 1, we show all the sociodemographic variables of the sample.

### 3.2. Bivariate Analysis

With regard to BMI, significant differences were found for the severity of OSAS (U = 393.5, *p* = 0.043); however, no significant differences were found between obese and non-obese individuals based on sex and the severity of obstructive sleep apnoea (X^2^ = 3677, *p* = 0.298). We analysed the possible differences between the AHI according to the presence or absence of type 2 diabetes mellitus in the sample. Significant differences were found in the AHI between the participants who had type 2 diabetes mellitus and those who did not (U = 937.5, *p* < 0.001), with a moderate/large effect size (biserial rank correlation = 0.449). The bivariate tests are presented in Table 2.

## 4. Discussion

The aim of this study was to determine whether type 2 diabetes mellitus in elderly people affected the level of severity of OSAS, obtaining significant differences in these variables; for instance, elderly people with diabetes mellitus had a higher degree of OSAS.

This is justified on the basis of the inherent changes in the ageing process. Thus, re-search such as that carried out by Edwards et al. [11] and Fallahi et al. [2] shows that ageing increases the risk of comorbid diseases for patients with type 2 diabetes mellitus, also worsening existing diseases, thereby causing adverse effects in the person’s sleep condition.

Authors such as Redline et al. [16], Reutrakul et al. [17], Subramanian et al. [6], and Sulit et al. [18] have shown that type 2 diabetes mellitus can affect the central control of breathing due to diabetic neuropathy, which compromises the function of the nerves responsible for respiratory regulation. In people with type 2 diabetes mellitus, altered neural reflexes in the upper airway can hinder the brain’s response to apnoeic episodes, making them more prolonged or frequent. Furthermore, diabetes-induced autonomic dysfunction can reduce the sensitivity of chemoreceptors responsible for detecting changes in oxygen and carbon dioxide levels, affecting the central nervous system’s ability to adjust breathing efficiently. This combination of factors contributes to the development and worsening of OSAS in diabetic patients [6,16,17,18].

The relationship between OSA and type 2 diabetes mellitus is bidirectional. Intermittent hypoxia, characteristic of OSA, generates increased sympathetic activity and oxidative stress. This stress causes chronic low-grade inflammation, which triggers the activation of the hypothalamic–pituitary–adrenal axis, increasing cortisol levels. Excess cortisol contributes to insulin resistance by interfering with normal insulin signaling, hindering cellular glucose uptake. Furthermore, frequent awakenings and sleep fragmentation affect the regulation of key hormones such as leptin and ghrelin, causing alterations in energy metabolism and increasing the risk of obesity, another factor that enhances insulin resistance. The dysfunction of pancreatic beta cells, responsible for insulin production, is exacerbated by systemic inflammation and hormonal imbalance, reducing their ability to maintain glycaemic homeostasis. Therefore, OSA not only impacts breathing during sleep, but also contributes to metabolic dysregulation, increasing the risk of developing type 2 diabetes mellitus and complicating its management in vulnerable populations such as older adults, who are also subject to changes associated with aging, such as the lengthening of the soft palate, structural changes, and increased fat deposition in the pharyngeal area [1,5,6,7,17,18].

The consumption of toxic substances such as tobacco and alcohol in elderly people with OSA may contribute to an increase in the severity of the disease and aggravate the risk of metabolic and cardiovascular complications. Smoking induces chronic inflammation in the airways, reducing the stability of the pharyngeal muscles and promoting their collapse during sleep, which increases intermittent hypoxia and oxidative stress. On the other hand, alcohol, by acting as a central nervous system depressant, causes excessive relaxation of the pharyngeal muscles, which increases airway obstruction and alters sleep architecture, increasing its fragmentation and reducing the time in restorative phases [19,20]. Both factors, together, contribute to the worsening of OSA in elderly people, promoting systemic inflammation, insulin resistance, and autonomic dysfunction, which increases the likelihood of developing or aggravating type 2 diabetes mellitus in this vulnerable population. Despite the scientific evidence found in the studies of Ioannidou et al. [19] and Mukamal [20], in the present study, no such association was found.

Regarding comorbid chronic diseases, OSA in older adults can be aggravated by the presence of chronic obstructive pulmonary disease (COPD) and cardiovascular diseases, generating a significant impact on respiratory and metabolic health. The coexistence of OSA and COPD, known as “overlap syndrome,” is associated with greater nocturnal hypoxia, systemic inflammation, and autonomic dysfunction, which increases the risk of cardiovascular complications. Furthermore, OSA contributes to the development of arterial hypertension, heart failure, and arrhythmias due to the activation of the sympathetic nervous system and the oxidative stress generated by intermittent hypoxia [21,22]. Although no relationship between these variables was found in the present study, Milicic et al. [21] and van Zeller et al. [22] report that these effects can exacerbate the progression of cardiovascular diseases in older adults, increasing morbidity and mortality and reducing quality of life.

Subramanian et al. [6] found that patients with type 2 diabetes mellitus are 50% more likely to develop OSAS compared to people without diabetes mellitus, regardless of traditional risk factors or confounders of OSAS. In the mentioned study, the authors found incident predictors of OSAS in patients with type 2 diabetes mellitus, including the male sex, obesity, cardiovascular disease, and depression [6]; this is inconsistent with the results of our study, as no statistically significant differences were found between obese individuals and the severity of obstructive sleep apnoea based on sex.

With regard to sex, it is worth noting the existence of studies in which an association was found between excessive daytime sleepiness and glycaemic control in men with obesity; no such relationship was found in the worsening of OSAS in non-obese diabetic men or women of any BMI category in the present study. In contrast, other studies that included mostly women with type 2 diabetes reported higher scores on the daytime sleepiness scale with higher HbA1c levels [23,24].

On the other hand, with regard to the age variable, authors such as Alshehri et al. [24], Edwards et al. [11], and Fallahi et al. [2] have shown that the prevalence of OSAS tends to increase with age in people with type 2 diabetes, although in the present study, no significant relationship was found [2,11,24]. The lack of a relationship between age and the incidence of OSAS in patients with type 2 diabetes is explained by the fact that the main studies reviewed did not take into account the age variable, as they included an adult population aged 18 years and older. There is only one epidemiological study that examines a large cohort of healthy elderly people, which found OSAS as a risk factor for developing type 2 diabetes, although without considering the severity of the condition [25]; otherwise, only the present study has contributed this information.

Aronsohn et al. [10] found a clear and graded inverse relationship between OSAS severity and glucose control in patients with type 2 diabetes mellitus, after controlling for confounding factors, such as degree of adiposity [10]; however, this and subsequent studies have been carried out in young and middle-aged adults, leaving out the elderly, or including them with middle-aged adults [5,8,9,10,23,26]. In the case of the research by Aurora et al. [23], the association between severe OSAS and type 2 diabetes mellitus was found only in people with excessive daytime sleepiness [23], while in the study by Byun et al. [5], this association was established with sleep fragmentation, oxygen desaturation, and anatomical dysfunction [5]. On the other hand, studies have found no association between OSAS severity and microvascular endothelial dysfunction in patients with type 2 diabetes. The lack of association in the aforementioned studies may be due to the short PSG recording (4 h) in some of these investigations, which is insufficient to detect an association between OSAS severity and type 2 diabetes [9,27].

In line with this, authors such as Aronsohn et al. [10] and Duc et al. [28] support the hypothesis that a reduction in OSAS severity may improve glycaemic control, thereby reducing the number of drugs taken, contributing to the improvement of millions of patients with type 2 diabetes [10,28]. Previous work has demonstrated the efficacy of CPAP in people with and without type 2 diabetes mellitus depending on the obesity of the individual [23]. After the sustained use of CPAP for a period of 6 months, improvements in nocturnal glucose levels and increased insulin sensitivity were found after 3 months with CPAP [23,29,30,31,32,33,34]. In the present investigation, a relationship was found between these variables and CPAP use.

There are studies that advocate proper weight control, since avoiding obesity im-proves insulin resistance caused by increased plasma adiponectin levels in OSAS [26]. Pharmacotherapy in type 2 diabetes, such as sulphonylureas, glitazones, and insulin, contributes to undesirable consequences like weight gain or the exacerbation of the severity of existing OSAS, thus increasing cardiovascular risks [6,10]. In this regard and consider-ing that elderly patients are generally polymedicated as a result of associated comorbidi-ties, they may also present with sleep pattern disturbance as an adverse effect [3].

Given the high prevalence of OSAS in people with type 2 diabetes mellitus, the International Diabetes Federation (IDF) considered screening people with diabetes for OSAS. However, due to the large number of patients with type 2 diabetes mellitus and the high cost of diagnostic methods for OSAS, this recommendation has proved difficult to implement [6].

Therefore, and as a result of the discrepancies observed in the published studies, given the sample selection bias due to the heterogeneity of the sample, the variability of severity due to the use of different diagnostic methods, the duration of the disease, the wide age range, and the non-consideration of the elderly, it was difficult to make comparisons with other studies based on the results obtained in the present investigation.

This study has strengths and limitations that must be pointed out. On the one hand, elderly people aged 65 years and over were considered as the only population group in order to be able to treat this population without discrimination, and the diagnosis of OSAS was obtained through the gold standard (PSG); on the other hand, the main limitation of the study is its cross-sectional design.

Thus, it is essential to conduct future research with a large cohort of elderly subjects, followed longitudinally, in order to assess how the ageing process alters the factors that cause these diseases, as well as their sequelae.

## 5. Conclusions

OSAS in the elderly and its relationship with type 2 diabetes mellitus presents several gaps in the scientific literature. Although it has been established that OSAS affects glucose metabolism, the specific pathophysiological mechanisms in geriatric populations have not been fully defined. Furthermore, the impact of CPAP treatment on glycaemic control in the elderly remains a subject of research, especially regarding its long-term effectiveness. Identifying independent predictors of diabetes in patients with OSAS requires more robust studies to validate these risk factors. Furthermore, the underdiagnosis of OSA in elderly patients with type 2 diabetes mellitus and the scarcity of longitudinal studies hinder our understanding of the progression of both diseases in this population. More in-depth research into these aspects is crucial to optimize prevention and treatment strategies.

## Figures and Tables

**Table 1:** Characteristics of the sample.

Variable	Categories	N (%)
Sex	Men	76 (56.72)
	Women	58 (43.28)
Level of education	Illiterate	22 (16.42)
	Basic	87 (64.93)
	Baccalaureate	20 (14.93)
	University	5 (3.73)
Marital status	Single	3 (2.24)
	Married	107 (79.85)
	Divorced	2 (1.49)
	Widowed	22 (16.42)
CPAP use	Yes	61 (45.52)
	No	73 (54.48)
Diabetes mellitus	Yes	34 (25.37)
	No	100 (74.63)
Tobacco smoker	Yes	62 (46.27)
	No	72 (53.73)
Alcohol drinker	Yes	25 (18.66)
	No	109 (81.34)
Chronic obstructive pulmonary disease	Yes	46 (34.33)
	No	88 (65.67)
Coronary heart disease	Yes	66 (49.25)
	No	68 (50.75)
BMI category	Normal	10 (7.46)
	Obesity	124 (82.54)
Severity of obstructive sleep apnoea	No apnoea	58 (43.28)
	Mild apnoea	28 (20.9)
	Moderate apnoea	21 (15.67)
	Severe apnoea	27 (20.15)

**Table 2:** Bivariate contrast before the intervention.

Variable	Category	M ± SD	Contrast	*p*-Value
Sex	Men	1 ± 1.13	U = 1912	0.167
	Women	1.29 ± 1.23		
Age			Rho = 0.120	0.167
CPAP use	Yes	0.44 ± 0.79	U = 3654	<0.001 *
	No	1.76 ± 1.12		
Diabetes mellitus	Yes	1.85 ± 1.16	U = 937.5	<0.001 *
	No	0.88 ± 1.09		
Tobacco smoker	Yes	0.86 ± 1.13	U = 2776.5	0.010
	No	1.36 ± 1.18		
Alcohol drinker	Yes	0.92 ± 1.29	U = 1567	0.219
	No	1.17 ± 1.15		
Chronic obstructive pulmonary disease	Yes	1 ± 1.19	U = 2224	0.324
	No	1.19 ± 1.17		
Coronary heart disease	Yes	1.08 ± 1.21	U = 2372	0.549
	No	1.18 ± 1.16		
BMI category	Normal	0.4 ± 0.7	U = 393.5	0.043 *
	Obesity	1.18 ± 1.19		

* Statistically significant differences; M: Mean; SD: Standard deviation.

## Data Availability

All data generated or analysed during this study are available at: https://doi.org/10.7910/DVN/LLJM2U.
